# Apocrine Breast Cancer: A Case Report

**DOI:** 10.7759/cureus.57789

**Published:** 2024-04-07

**Authors:** Soumiya Samba, Ahmed Bensghier, Soufiane Berhili, Mohamed Moukhlissi, Loubna Mezouar

**Affiliations:** 1 Department of Radiation Oncology, Mohammed VI University Hospital, Faculty of Medicine and Pharmacy, Mohammed First University, Oujda, MAR

**Keywords:** malignancy, breast cancer, chemotherapy, radiation therapy, breast apocrine carcinoma

## Abstract

Breast cancer is the most frequent malignancy among women worldwide, including a wide range of histological subtypes, from typical expressions like invasive ductal carcinoma to less common variations like apocrine breast carcinoma. This document discusses the case of a 65-year-old female with apocrine breast cancer, who presented with a chronic mastodynia. This case highlights the importance of being aware of apocrine breast cancer.

## Introduction

Apocrine carcinoma (AC) is an uncommon form of breast cancer, representing around 0.5-4% of all cases of invasive breast carcinoma in women [1,2].

Under microscopic examination, AC has a similar structural development pattern to that of invasive ductal carcinoma, particularly a variant identified as IDC-NOS (invasive ductal carcinoma, not otherwise specified). However, the main difference between the two is their cytological characteristics. The cells of AC have particular apocrine features, such as rich eosinophilic granular cytoplasm and prominent/multiple nucleoli. To date, there is no commonly acknowledged definition; thus, some pathologists confirm their diagnosis using staining methods that detect the presence of breast gross cystic disease protein 15 (GCDFP-15) in cystic disease fluid [3,4].

The apocrine epithelium in the breast is characterized by a lack of estrogen receptor (ER) and progesterone receptor (PR) expression while maintaining the presence of androgen receptor (AR) expression. Yet, there is inconsistency in the literature regarding the classification of these and other receptors in ACs [5].

The term "pure" AC refers to a distinctive cancer subtype in which more than 90% of tumor cells show apocrine morphology and lack expression of the ER and PR, regardless of HER2 status. This specific carcinoma type is associated with a lower rate of cancer-free survival in comparison to other histological types of breast cancer [6,7].

Initially, it was thought that the histological features of AC were similar to non-apocrine cancer [2]. However, ongoing discussions surround the outlook of AC, largely due to a significant number of cases being triple-negative, suggesting a potentially unfavorable prognosis [7,8].

In light of this, we intend to share our diagnostic attitude facing a 65-year-old female with a very specific morphological manifestation known as invasive apocrine breast cancer.

## Case presentation

We report the case of a 65-year-old female, a lifetime non-smoker, with a personal history of hypertension under well-balanced monotherapy and dyslipidemia under hygiene-dietetic measurement since the age of 50. She has no other personal or familial history. The patient has experienced a three-month history of chronic mastodynia in the right breast persisting. Despite this, there has been no occurrence of nipple discharge or skin retraction. Physical examination revealed the presence of a palpable 3-cm long-axis nodule in the upper outer quadrant of the right breast, mobile regarding both layers with no signs of inflammation. Lymph nodes and the contralateral breast were found to be normal.

**Figure 1:**
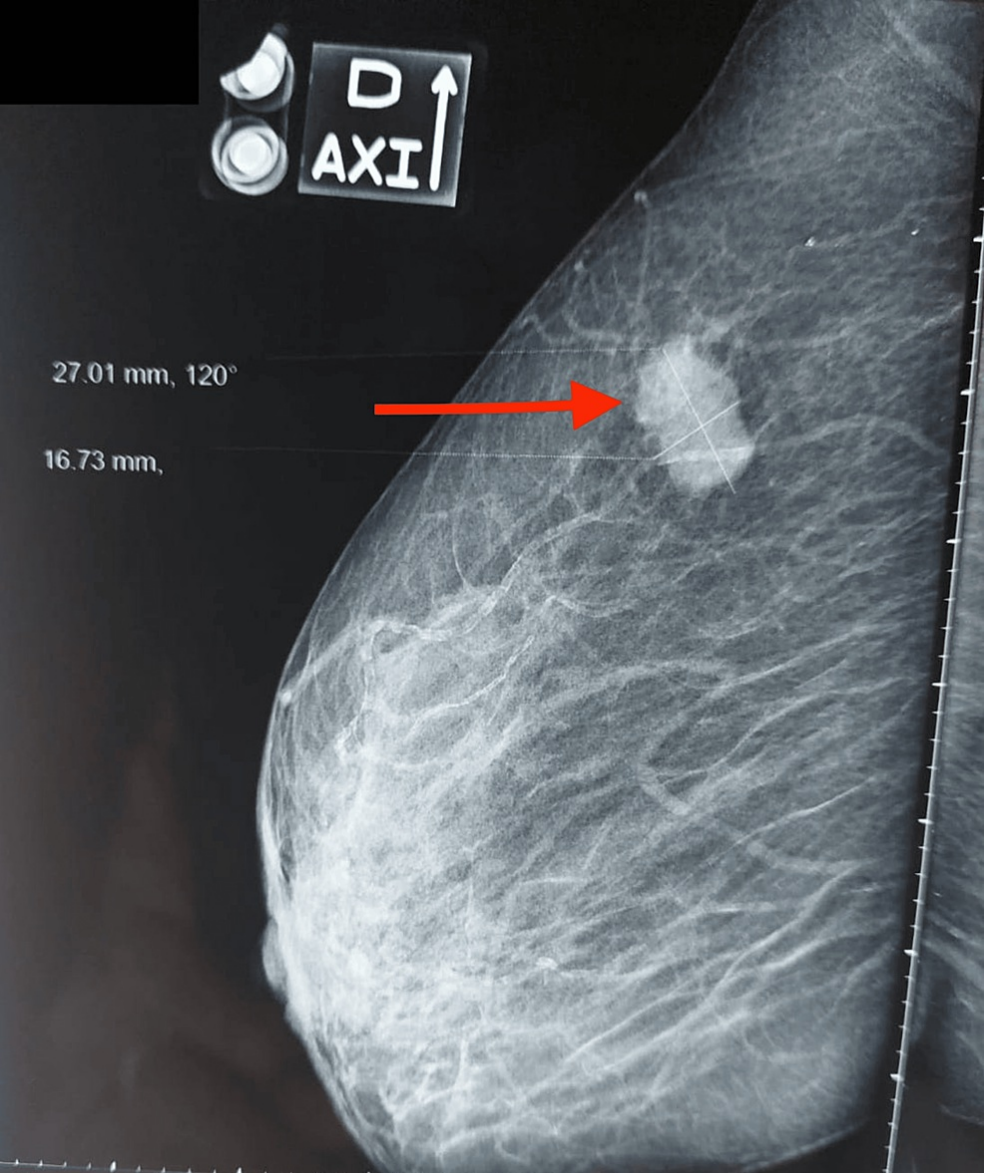
Mammogram view: a dense, fatty glandular breast lesion of type B exhibiting no skin thickening, along with opacity in the upper outer quadrant of the right breast displaying angular contours, categorized as BI-RADS 4c (red arrow) BI-RADS: Breast Imaging-Reporting and Data System

A bilateral mammogram showed a 27×17 mm suspect nodule in the upper outer quadrant of the right breast classified as ACR4 (Figure 1).

**Figure 2:**
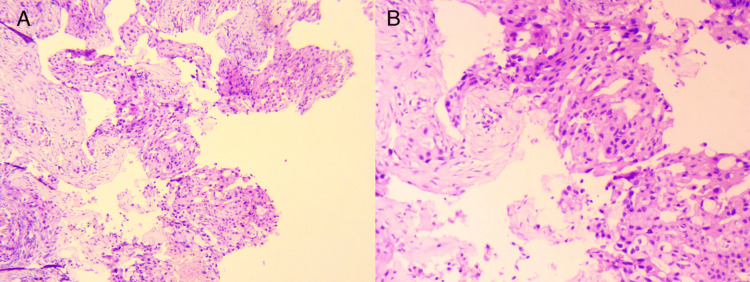
Histological imaging revealing a carcinomatous tumor proliferation composed of apocrine-type cells, characterized by abundant eosinophilic cytoplasm and hyperchromatic nuclei within a dense fibrous stroma

An ultrasound-guided fine-needle aspiration of the right breast mass was performed, leading to a histopathological diagnosis of AC. Immunohistochemical analysis revealed a lack of expression of ER and PR, while HER2 and AR were expressed positively (HER2 65% and AR 63%) (Figure 2).

**Figure 3:**
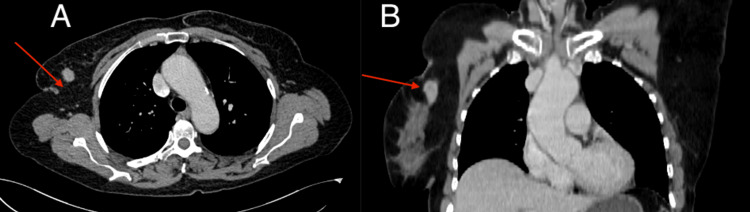
Chest CT scan with contrast injection revealing a right breast nodule in the upper outer quadrant (red arrows): (A) axial section and (B) frontal section

A CT scan of the thorax, abdomen, and pelvis was then conducted and showed a tumoral mass in the upper outer quadrant of the right breast, with axillary adenopathies of irregular contours, the largest measuring 20×25 mm (Figure 3).

Therefore, the decision was to realize a tumor excision of the right breast, accompanied by surgical axillary staging, which disclosed the presence of AC, classified as Grade II according to the Elston and Ellis classification [9]. The excised tumor measured 1.8 cm with no observation of in situ components or vascular emboli. Among the 17 dissected lymph nodes, seven exhibited metastasis. The tumor was categorized as pT1cpN2aM0 according to the TNM staging system.

After the surgical procedure, adjuvant chemotherapy was initiated, involving three rounds of anthracycline and cyclophosphamide, followed by three cycles of taxanes (paclitaxel) along with 17 doses of Herceptin. Anthracycline and cyclophosphamide are administered every 21 days for three days. Twenty-one days after the last administration of anthracycline and cyclophosphamide, paclitaxel is initiated and given every 21 days. The patient was also receiving Herceptin during the cycles of paclitaxel. After the final cycle of paclitaxel, the patient continued with Herceptin alone.

Four weeks after completing the last cycle of paclitaxel, the patient underwent adjuvant radiotherapy, specifically targeting the supraclavicular zone and right breast. The primary treatment involved a total dose of 42 Gy administered over 15 fractions of 2.8 Gy. Additionally, a tumor bed boost was applied, totaling 11.2 Gy distributed across four fractions of 2.8 Gy each [10]. The radiotherapy sessions were successfully completed without encountering any complications.

The surveillance was conducted via clinical examination and mammography every three months along with a CT scan once annually. The patient has maintained a disease-free status for the last two years, and there have been no reported instances of lymph node involvement or distant metastases during this period.

## Discussion

AC is an uncommon morphological subtype of invasive breast carcinoma in women, characterized by a predominantly or entirely apocrine-type epithelium. The exploration of this distinctive and infrequent disease is challenging, primarily due to the lack of consensus regarding its precise definition.

As per the World Health Organization (WHO) classification, a breast tumor is categorized as invasive apocrine breast cancer when a minimum of 90% of its cells display apocrine differentiation, featuring giant cells resembling sweat glands [6].

The prevalence of infiltrating apocrine cancer remains uncertain, primarily attributed to notable variations in its definition and consequently reported prevalence across different studies.

This entity is categorized among "relatively rare carcinomas" in specific series. Various studies have documented the prevalence of AC, with reported rates ranging from 0.5% to 4% of all invasive breast carcinomas in certain investigations. Conversely, other studies have indicated a prevalence of 1% [11,1].

At present, experts align on the notion that the definition of pure apocrine cancer should rely on the distinct appearance of cells and specific immunohistochemical features. This criterion closely mirrors the characteristics observed in normal apocrine epithelium [12]. Vranic et al. have proposed a comprehensive molecular characterization of "pure apocrine" breast cancer, defining it as a tumor expressing ARs while lacking ERs or PRs. Consistent with this paradigm, pure apocrine cancer may exhibit either HER2 overexpression or HER2 negativity. Thus, this delineation relies on both structural analysis and distinctive immunohistochemical properties, akin to those observed in normal apocrine epithelium [7].

Presently, the clinical and radiological manifestations of AC closely resemble those observed in invasive ductal carcinomas [3]. Clinically, AC often presents as a palpable tumor mass, occasionally accompanied by bloody nipple discharge, or, in some instances, as a cyst [13].

Due to the rarity of these cases, the optimal treatment for AC remains uncertain. Generally, they are approached in a manner similar to other subtypes of breast cancer. The primary treatment option involves surgery, which may entail breast-conserving surgery, mastectomy, and, if necessary, axillary surgery.

Furthermore, adjuvant treatment options such as hormonal therapy, radiotherapy, or chemotherapy are administered. The majority of these cancers are classified as triple-negative. HER2 and AR-positive patients have the option of receiving anti-HER2 and hormone therapy as part of their adjuvant treatment plan [14].

Mills et al. conducted a study involving 46 individuals diagnosed with breast apocrine cancer. Their findings suggest that AC is more frequently observed in older individuals, displaying smaller tumor size and a lower histopathological grade compared to other subtypes of triple-negative breast cancer [14]. Additionally, triple-negative apocrine breast carcinoma exhibits a more favorable overall survival (OS) outcome when compared to other forms of triple-negative breast malignancies [15].

Kaplan-Meier plots were employed to assess OS and disease-specific survival (DSS) in both histological subtypes. The analysis indicated that the prognosis for apocrine cancer is unfavorable. Additional investigations employing multifactorial analysis to assess risk variables impacting both OS and DSS have indicated that histological Grade II/III, tumor size exceeding 2 cm, and the presence of positive lymph nodes are associated with a poorer prognosis [16].

The prognosis of AC remains a subject of debate, with diverse outcomes observed in the conducted research thus far. Some types of invasive carcinomas exhibit superior survival rates, while others demonstrate comparable prognoses when compared to each other [7].

## Conclusions

Apocrine breast cancer, despite its rarity, presents a unique opportunity to explore novel and personalized treatment approaches. A more refined categorization of subgroups, considering ARs and other distinct molecular markers, is essential. Additionally, understanding how these markers influence therapy choices is crucial for advancing targeted and effective treatment strategies.
